# Psychometrics validation of the Chinese version of social support for exercise scale among adolescents in China

**DOI:** 10.1371/journal.pone.0299497

**Published:** 2024-06-20

**Authors:** Liying Yao, Ke Zhou, Yanli Zhou, Yee Cheng Kueh, Tingyu Xu, Mingzhu Pan, Anwar P. P. Abdul Majeed, Zhongbiao Liu, Garry Kuan

**Affiliations:** 1 School of Physical Education, Shangrao Normal University, Jiangxi, Shangrao, China; 2 Exercise and Sports Science Programme, School of Health Sciences, Universiti Sains Malaysia, Kubang Kerian, Kelantan, Malaysia; 3 Sports Reform and Development Research Center of Henan University, School of Physical Education, Henan University, Henan, China; 4 Biostatistics and Research Methodology Unit, School of Medical Sciences, Universiti Sains Malaysia, Kubang Kerian, Kelantan, Malaysia; 5 College of Physical Education and Health, Nanchang Institute of Science and Technology, Jiangxi, Nanchang, China; 6 School of Robotics, XJTLU Entrepreneur College (Taicang), Xi’an Jiaotong-Liverpool University, Suzhou, China; 7 School of Physical Education & Sports Science, South China Normal University, Guangzhou, China; 8 Department of Life Sciences, Brunel University, London, United Kingdom; Tsinghua University, CHINA

## Abstract

Physical activity (PA) is widely recognized as crucial for human health, yet the low level of PA in adolescents continues to raise major concerns. This study aims to validate the Chinese version of the Social Support Scale for Exercise (SE) and establish its reliability among Chinese adolescents. A cross-sectional study was conducted on two primary and two secondary schools in central China. Students were recruited using a random cluster sampling method, and written informed consent was provided after they were briefed on the purpose of the study. The standard forward-backward translation was applied to translate the English version of the SE into Chinese. The Social Support Scale used in this study consists of two factors: family support and friend support. Data were analyzed using Mplus 8 for the CFA, composite reliability (CR), average variance extracted (AVE), and intra-class correlation coefficients (ICC) were calculated. A total of 1422 students (boys = 838, girls = 604) with a mean age of 11 years (SD = 1.6) participated in the study. The measurement model of the translated social support scale fit the data well: CFI = .935; TLI = .929; SRMR = .038; RMSEA = .053, with a 90% confidence interval of (.051, .056; RMSEA *p* < .001). The composite reliability values of .935 for family support and .948 for friend support were acceptable. The intra-class correlation coefficients (ICC) based on test-retest were .928 for family support, and .904 for friend support. Hence, the Chinese version of the SE was valid and reliable, its implementation will provide researchers with a valuable tool to comprehensively assess Chinese adolescents’ exercise-related social support and help develop targeted and effective interventions to improve their physical activity levels.

## Introduction

Physical activity (PA) is considered a vital factor affecting human physical and mental health, especially for adolescents. Existing research indicates that PA contributes greatly to the health and personal development of adolescents, as well as to the prevention of chronic diseases such as hypertension, diabetes, and cardiovascular disease [1]. However, in 2016, 81% of adolescents worldwide did not meet the PA level recommended by the World Health Organization [2], demonstrating that physical inactivity is not just pervasive in developed countries but is also rapidly increasing in developing countries.

Extensive effort has been directed toward comprehending the factors that influence individual participation in PA. Among these endeavors, Bronfenbrenner’s Social-Ecological Model (SEM) presents a multidimensional interaction framework that has been widely used to understand the influences on PA participation [3]. The model argues that changes in individual behavior are influenced not only by intrapersonal factors such as age and gender but also by interactions with the social and environmental contexts in which individuals live [4]. SEM notably highlights the crucial role of social support in motivating and maintaining PA, particularly among adolescents [1, 5]. Social cognitive theory further proposes that social support may act as a determinant of adolescents’ PA engagement, influencing various aspects of their decision-making regarding PA, from getting started to sticking with it or stopping altogether [6]. Enhanced social support is recognized as pivotal for enhancing self-efficacy and nurturing both new and active exercisers, thereby effectively catalyzing PA [7, 8].

Different notions and categorizations have been used to clarify the various dimensions of social support. While its conceptualization varies across research domains, three consistent attributes endure: it involves assistance and aid, relies on external interpersonal networks, and encompasses both moral and tangible backing [9]. Researchers have also classified social support according to its source and type. As the primary source of social support, parents, family members, friends, and teachers were the most widely surveyed subjects [10]. Moreover, the type of social support could be varied depending on the characteristics of the sports in which people participated [11]. For instance, parents often provide support by acquiring sports gear, facilitating transportation to sports venues, and offering encouragement for leisure-time exercises. In contrast, support from friends typically involves engaging in more rigorous sports or physical activities together. It has been established that adolescents who perceived a more comprehensive range of social support were more likely to engage in more physical activities [12].

Given the importance of social support, it is also seen as a crucial component of health behavioral interventions to promote PA. Hence, effective and reliable instruments to measure social support for PA in diverse populations are needed in the research community. To address this need, scholars from all over the world have made continuous efforts. In 1987, the original Social Support Scale for Exercise (SE) was developed by Sallis and his colleagues [13]. This scale was initially constructed from three subscales: Family Involvement, Family Punishment, and Friend Involvement. It was then revised to two subscales: Family Support (15 items) and Friend Support (5 items), and validated as a reliable instrument among 171 college students with acceptable test-retest reliabilities of the factors (range, γ = 0.55–0.86) and high internal consistencies (range, α = 0.61–0.91). The SE has been widely acknowledged as an effective instrument among the English-speaking population [14].

These subscales were then translated into different languages and introduced in different countries and populations [15–17]. Nevertheless, since the number of items in the original scale is unbalanced across factors, this has the potential to weaken the scale’s psychometric properties and limit its future applicability and generalizability. Thus, some translated versions have also modified the number of items in the scale. Korean scholars Yang et al. modified the original SE scale to a two-factor 24-item version (12 items for family support and 12 items for friend support) when they introduced it into Korea, and this version has been supported by other scholars [3]. However, as of now, no research has been conducted on this version within China. Furthermore, Validation studies of exercise-related social support scales remain limited [18, 19], particularly among adolescent populations. This gap in research clearly does not meet the needs of practical applications.

China has recently enacted several major policies, such as the "Regulations on National Fitness," "Healthy China 2030," and the "Youth Physical Activity Promotion Program." These policies not only outline specific requirements and recommendations for promoting adolescent health and PA but also foster an environment conducive to enhancing the physical fitness of adolescents [20, 21]. Given these policy developments, there is a clear demand for well-established measurement tools that can aid researchers in gaining a deeper understanding of the current state of social support for adolescent sports participation in China, as well as the associated challenges. This understanding is crucial for the development of effective strategies aimed at further increasing adolescents’ engagement in PA. Therefore, the primary objective of this study is to assess the reliability and validity of the Chinese version of the SE (SE-C) among Chinese adolescents. The validity of the questionnaire was examined by using confirmatory factor analysis (CFA) and the reliability was determined by using internal consistency reliability and test-retest reliability. Establishing the validity and reliability of SE-C will enable future researchers, health educators, and sports psychologists to use the scale to measure the level of social support for exercise among adolescents in China.

## Methods

### Participants

This sample consists of 1422 Chinese students from Shangrao, Jiangxi Province, in central China. A larger sample typically yields more precise and reliable results [22]. Therefore, a sample size of 1422 was sufficient to conduct confirmatory factor analysis (CFA) on the 24-item social support scale in this study. We divided Shangrao city into 2 parts (Downtown and suburbs), and a multistage, random cluster sampling method was adopted. One primary school and one middle school were randomly selected from each part. Then, four primary and middle schools were investigated. Participants were aged from 9 to 15 years old (Mean age = 11 years old, SD = 1.6). Among all the sample sizes, 838 (58%) were boys and 604 (42%) were girls. In terms of degree, there were 738 primary school students (51.2%) and 704 middle school students (48.8%).

### Questionnaire translation

Questionnaire translation is one of the most crucial processes in researching a sample of individuals whose culture or language differs from that of the original group. The appropriateness of translation is directly related to the accuracy of the research results. Permission to use the questionnaire was obtained by emailing the original author. The original scale was translated into Chinese utilizing Brislin’s (1986) standard forward-backward translation procedure to improve acceptance and comprehension of the translated version [23]. Firstly, two bilingual researchers with no experience in sports psychology translated the original SE scale into Mandarin. The authors then updated the translation to fit the professional background and terminology. Secondly, five experts with professional backgrounds in Sports Science, Sports Psychology, and Physical Education were invited to assess the forward translation and discuss a better version. Furthermore, another two bilingual researchers were invited to back-translate the improved Chinese version of the social support scale for exercise into English and compare it with the original scale [24]. Changes are made until an appropriate Chinese version is achieved. To confirm cross-cultural adaptation and the clarity of the version, Ten Chinese-speaking students, aged 9 to 12, reviewed the questionnaire for clarity and comprehension. Their feedback, focused on brevity and clarity, informed modifications to the final version. Subsequently, another group of ten native Chinese-speaking students in the same age range confirmed the questionnaire’s clarity with no new concerns raised. The final version was then finalized.

### Measures

#### The demographic and sports activities information

The demographic information was reported as means and standard deviations (SD) or as frequencies and percentages. Including age, gender, grade, height, weight, sports participation, and time spent per week doing PA or exercise.

#### The social support scale for exercise

The social support scale for exercise utilized in this study originated from Sallis et al. [13]. It was initially a five-point Likert scale comprising two subscales with a total of 20 items: family support (15 items) and friend support (5 items). This scale has been widely acknowledged as a reliable tool for assessing social support for exercise. Subsequently, Korean researchers refined and expanded it to include 24 items (12 items measuring family support and 12 items measuring friend support) [25], Participants rated perceived support from family or friends (e.g., "encourages me to stick to my exercise program," "praises me for the changes in my body after I exercise") on a 5-point scale from 1 (never) to 5 (very often). Therefore, the Chinese version of the Social Support for Exercise Scale (SE) was derived from the updated version by Yang et al. (2005), with reported Cronbach’s alpha values of .85 for family support and .88 for friend support, and test-retest values of .83 for family support and .89 for friend support [25].

### Data collection

The current study was an international collaboration involving scholars from China, Malaysia, and Korea and was conducted in full compliance with local systems. We obtained ethical approval from the Universiti Sains Malaysia Human Research Ethics Committee (Approval No: USM/JEPeM/21090638) and the Jiangxi Medical College Human Research Ethics Committee (Approval No: (RH)2022-5), adhering to the principles of the Declaration of Helsinki. This study was a cross-sectional study that used a self-reported questionnaire in four primary and middle schools in China. Data collection was performed from March to April 2022 in Shangrao, a city located in central China.

During the data collection phase, after thoroughly explaining the study’s purpose and procedures to the participants, the researcher provided them with informed consent and parental consent forms to take home and discuss with their parents or guardians. Only students who returned the signed informed consent and parental consent forms the following day were allowed to fill out the questionnaires. The inclusion criteria were as follows: (1) students aged 9 to 15 in primary and middle schools; (2) students who understand the information explained by the researcher and agreed to participate. Students with mental disorders or physical disabilities that stopped them from being active were excluded. The average time required to complete the questionnaire was approximately 15 minutes.

A total of 1,485 questionnaires were collected, and 1,422 of those were returned to the researchers with full answers to each item. Accordingly, the final sample size was 1422 with no missing values. To evaluate the retest reliability of the scale, 76 participants resubmitted the questionnaire on day 14 for a second time.

### Statistical analysis

Statistical analysis was conducted using SPSS Version 27.0 [26] and Mplus Version 8 [27]. Data were checked for missing values before analysis, and only fully answered questionnaires were included in the analysis. Confirmatory factor analysis (CFA) was applied using Mplus 8 to evaluate the validity of the initial measurement model (two factors with 24 items). All 24 items were considered as observable variables in the CFA analysis, while the factor(s) were treated as the latent variable (s). The MLR Estimator was chosen due to its ability to handle non-normal data distributions and produce robust estimates, including a mean-adjusted chi-square statistic along with standard errors [27].

A standardized factor loading threshold of .40 or higher was utilized to determine satisfactory factor loading for all items. This criterion was employed to decide whether to retain or exclude an item from the analysis [28]. A combination of fit criteria has been suggested to evaluate the model fit [29]. These model fit criteria include the Bentler–Bonnet Comparative fit index (CFI), Tucker and Lewis index (TLI), Root means square error of approximation (RMSEA), probability RMSEA, and standardized root mean square residual (SRMR). The model is fit if the CFI and TLI are greater than .90, the RMSEA is less than .07, the RMSEA *p*-value is less than .50, and the SRMR is less than .08.

In CFA, assessment of construct validity among item measures entailed evaluating both convergent validity and discriminant validity. Convergent validity quantified shared variance among items within a specific construct [28], using factor loadings, Average Variance Extracted (AVE), and reliability as measures [30]. To address reliability, Cronbach’s alpha which is frequently reported for internal consistency [28], composite reliability (CR) was advocated due to its suitability when accounting for residual covariances [31] were reported. The recommended threshold for Cronbach’s alpha is ≥ .70 [28], CR is ≥ .70, and ≥ .50 for AVE [32].

Discriminant validity was determined through the assessment of correlations between model factors. Adequate discriminant validity is established when factor correlations are less than or equal to .85 [30]. Test-retest reliability was examined using a sub-sample of 76 participants to calculate the intra-class correlation (ICC). Reliability was deemed satisfactory when ICC values exceeded .70 [33].

## Results

### Characteristics of participants

The participants in this study ranged in age from 9 to 15 years old, with an average age of 11 years (SD = 1.6). Out of the total sample, 838 (58%) were boys, and 604 (42%) were girls. Regarding their educational level, there were 738 students (51.2%) from primary schools and 704 students (48.8%) from middle schools. Most students (99.3%) participated in PA an average of 2 days per week for an average of 30 minutes each time. The major physical activities reported by participants included basketball, table tennis, jogging, badminton, rope skipping, brisk walking, bicycling, swimming, and soccer.

### Social support scale for exercise (SE) measurement model

**Fig 1:**
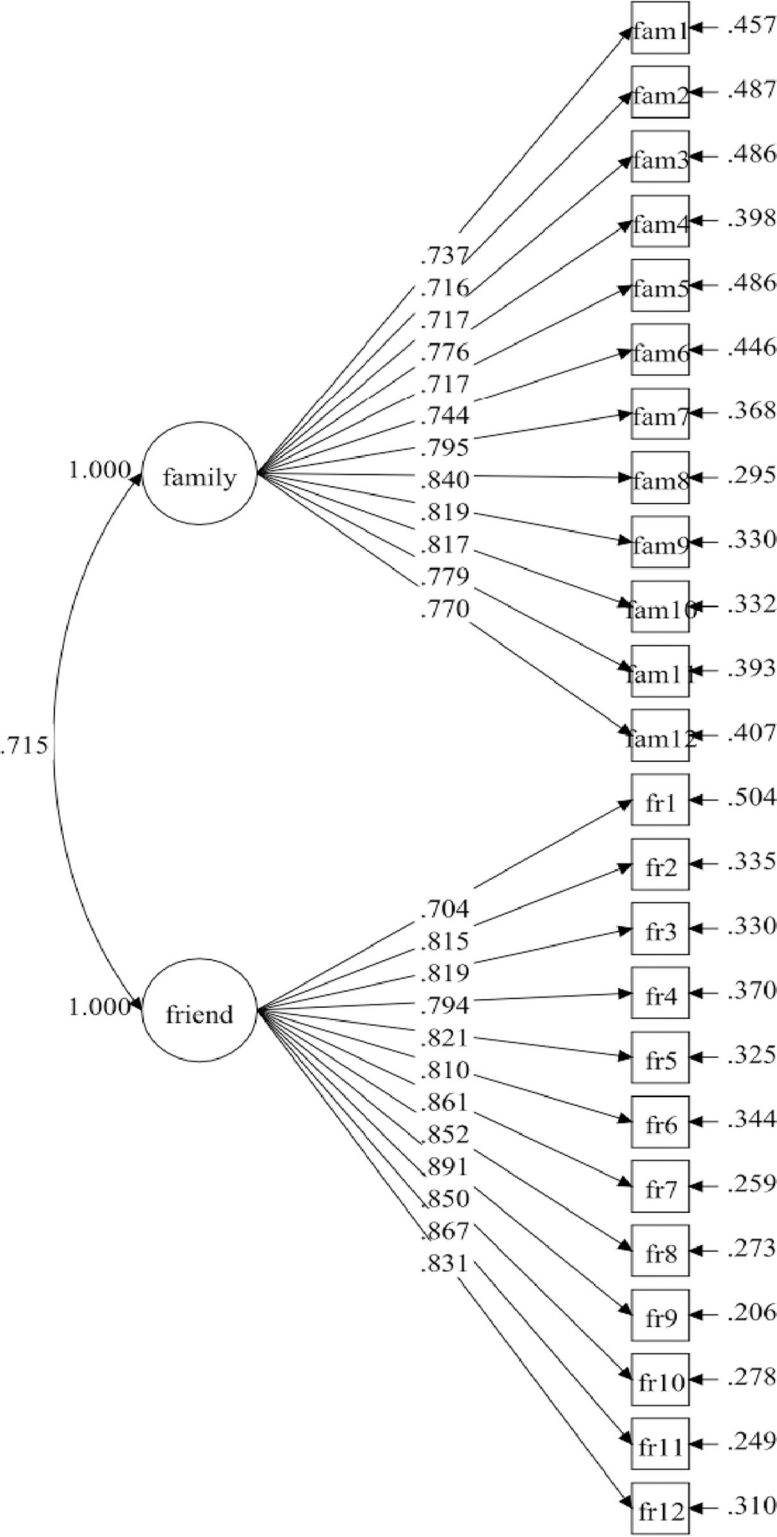
Social support measurement model (Model-1).

According to previous studies [25], the original measuring model for social support included two factors with a total of 24 items: family support (12 items) and friend support (12 items). The tests of the Chinese version of the measurement model (Model-1) demonstrate excellent match indices with the data: CFI = .935; TLI = .929; SRMR = .038; RMSEA (CI: 90%) = .053 (.051, .056; RMSEA *p* < .001 (Table 1). All fit indices are within the acceptable value range. the standardized factor loadings within the model also showed very good results ranging from .70 to .89 with a *p*-value < .001 (Fig 1).

**Table 1:** Summary for social support model fit indices.

Path Model	RMSEA (90%CI)	CFI	TLI	SRMR
Model-1	.053(.051, .056)	.935	.929	.038
Model-2	.051(.047, .054)	.933	.927	.040
Model-3	.055(.053, .058)	.928	.921	.039

Notes: RMSEA = Root Mean Square Error of Approximation; CI = Confidence Interval for relevant point estimates; CFI = Comparative Fit Index; TLI = Tucker Lewis Index; SRMR = Standard Root Mean Square Residual; Model-1 = all sample group; Model-2: sample from primary school students; Model-3: sample from middle school students.

**Fig 2:**
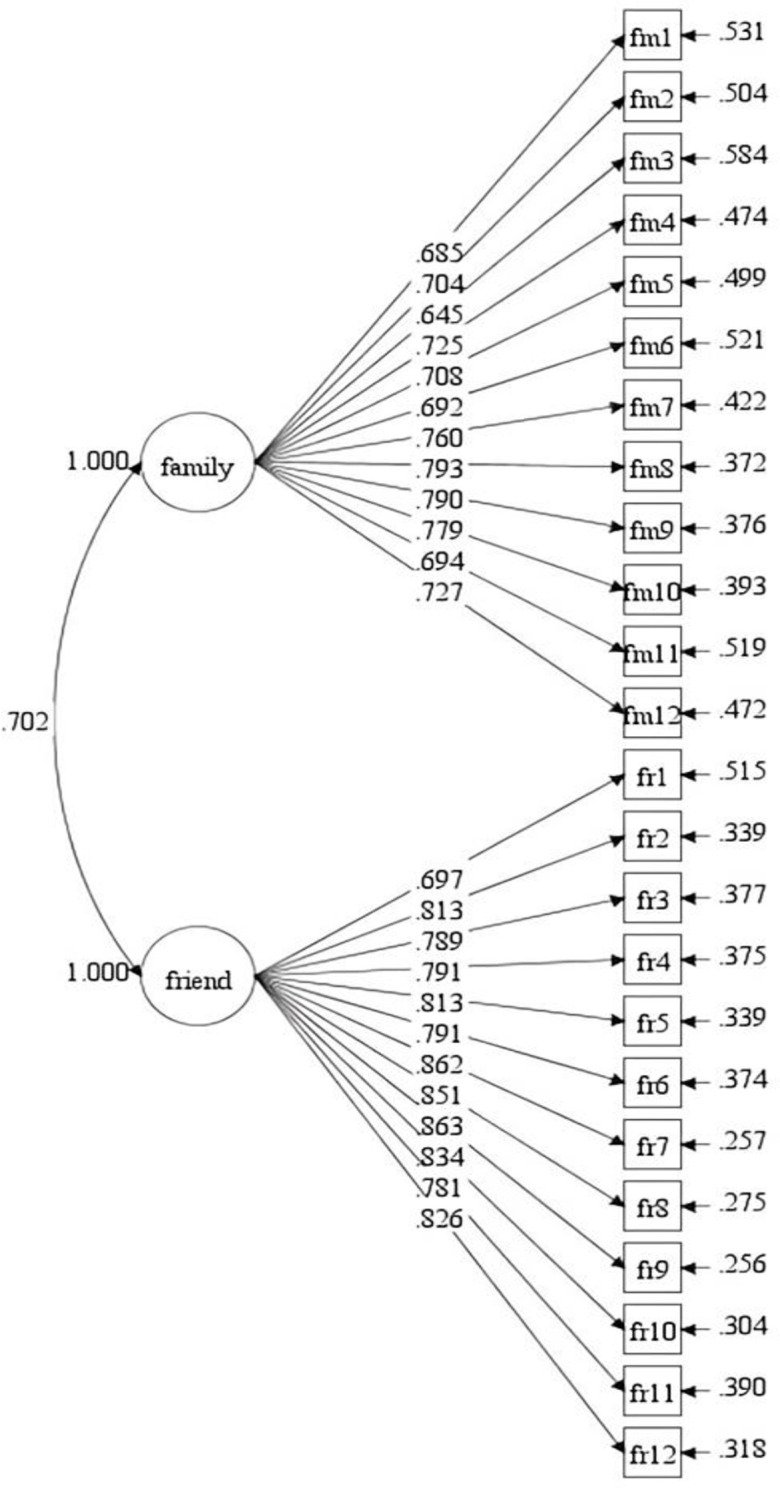
Social support measurement model (Model-2).

**Fig 3:**
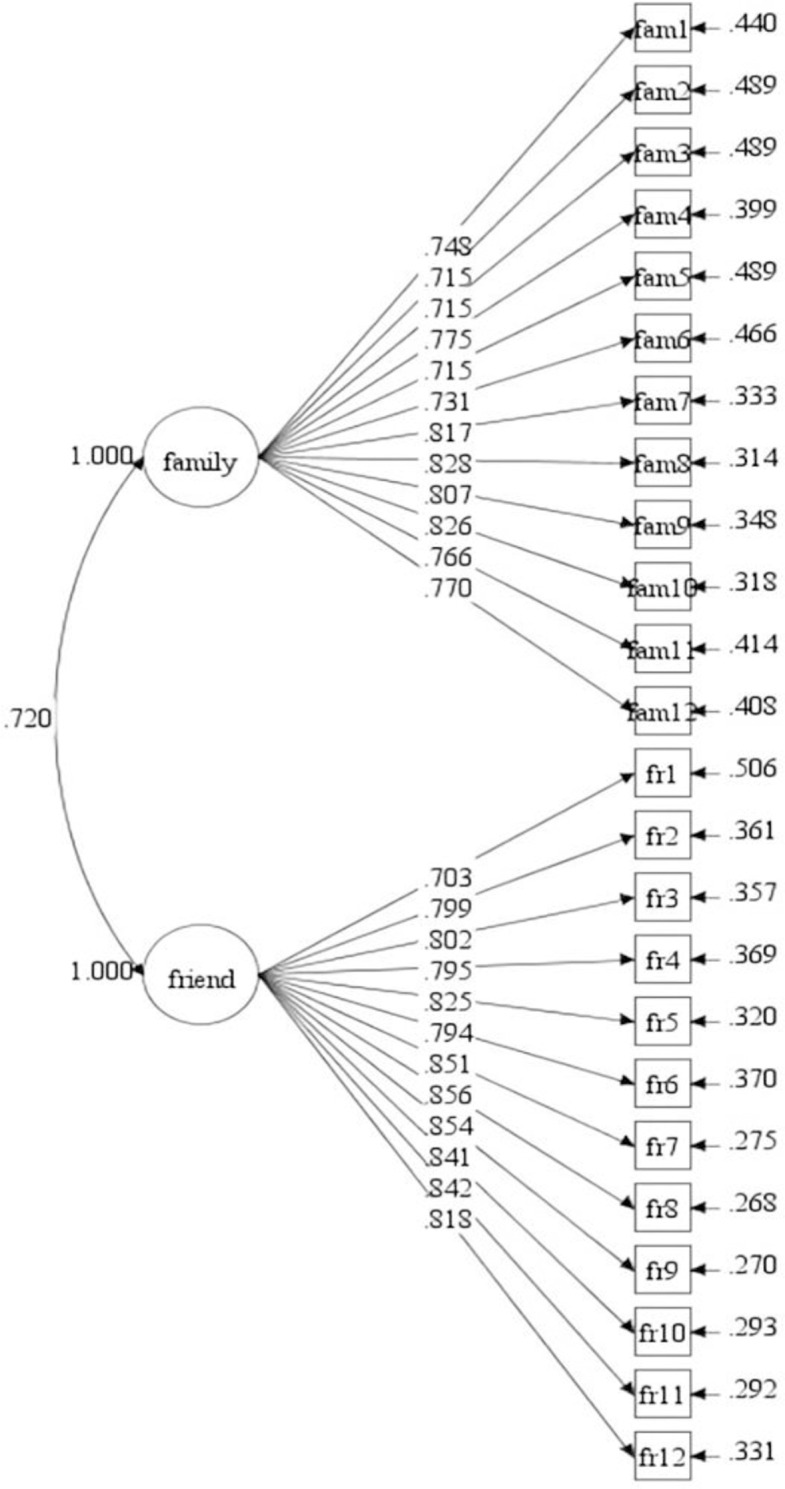
Social support measurement model (Model-3).

Meanwhile, the participants in our study included children and adolescents in primary and middle schools. To further determine the replicability of the Chinese version of the SE among students in different academic levels, we divided the participants into a primary school group (Model-2) and a middle school group (Model-3) on the basis of the overall sample (Model-1) and tested the models’ fitness individually. The CFA results revealed that the model adequately matched the data for both age groups (see Table 1, Figs 2 and 3).

### Composite reliability (CR) and discriminant validity

The CR values of the specified model (Model-1) were .935 for family support and .948 for friend support. The AVE values were .545 and .621, respectively. Both CR and AVE values exceeded the recommended thresholds of .60 [32] and .50 [30]. Additionally, the factor correlations between the family support factor and the friend support factor were .715 for Model-1, .702 for Model-2, and .720 for Model-3, all statistically significant but under .85, indicating high discriminant validity [31]. Thus, the model achieved adequate convergence and discriminant validity, as indicated by the CR, AVE, scale factor loading, and standardized factor covariance. Table 2 presents the CR, AVE values, and the factors correlation coefficient of the Model-1, Model-2, and Model-3.

**Table 2:** Composite reliability (CR), average variance extraction (AVE), and factor correlation for social support Model-1, -Model-2, and Model-3.

Variables	CR	AVE	Family support
Model-1 (All sample)			
Family support	.946	.593	-
Friend support	.963	.685	.715*
Model-2 (Primary school)			
Family support	.930	.528	-
Friend support	.958	.657	.702*
Model-3 (Middle school)			
Family support	.945	.591	-
Friend support	.958	.654	.720*

Note: *Correlation is significant at the .001 level (two-tailed)

### Test-retest reliability

During a two-week retest reliability check, intra-class correlation coefficients (ICC) were obtained for 76 participants and showed appropriate stability. On the social support scale, their mean score dropped from 68.3 to 66.8 (Day 1 (SD = 18.3) to Day 14 (SD = 19.1). The ICC value was .928 for family support (95% CI = .886 - .954, *p*-value < .001), and .904 for friend support (95% CI = .848 - .939, *p*-value < .001).

### Internal consistency

The Cronbach alpha of Model-1 was .966, .960 for Model-2 (Primary school), and .969 for Model-3 (Middle school), respectively. According to Mallery and George (2000), Cronbach alpha values of > .70 were considered acceptable, while alpha values greater than .90 were considered excellent outcomes [26].

## Discussion

In this study, the researchers translated the English version of the social support scale for exercise into Chinese and then examined its validity and reliability among Chinese primary and middle school students using the confirmatory factor analysis. The Chinese version of the SE revealed adequate validity and reliability with the observed data. The scale was well suited to the data and presented considerable proof for structure validity.

With the study sample, the Chinese version of the social support models validated in the present study demonstrated adequate internal consistency. The Cronbach’s alpha values of .966 for Model-1, .960 for Model-2 (primary school), and .969 for Model-3 (middle school) are consistent with previous research [25], where values of .85 for family support and .88 for friend support were reported. These values suggest a stable and consistent structure among both primary and middle school students in China [14]. Furthermore, all item-total correlations exceeded the threshold of .30, indicating that each item significantly contributed to its corresponding core factor measurement, thus establishing adequate internal reliability for the scales. Additionally, our study observed all retest ICC values to be above .90, reflecting a high level of stability in the scales. These findings are consistent with earlier results reported by Yang et al. (test-retest values of .83 for family support; and .89 for friend support), further reinforcing the scales’ reliability [33].

To evaluate the validity of the measurement model, the factor model of social support was investigated and confirmed by CFA. The social support scale for exercise was designed with two factors and 24 items. As no items were removed from the initial version, the results of the CFA indicated that all models matched the data of Chinese adolescents quite well. Based on the CR and AVE evaluations, the reliability of the models exceeded the suggested levels of .60 and .50. respectively [30, 32]. It implies that each model has consistent estimation precision and sufficient convergent validity [34]. Further, all correlations between variables in the three models were lower than 0.85, indicating strong discriminant validity. Thus, the model met the convergent and discriminant validity criteria regarding scale item loadings, CR, AVE, and standardized factor covariates.

Extensive research consistently underscores the significant role of psychological factors, such as social support and self-efficacy, in shaping children’s engagement in PA [35]. These findings remain consistent across diverse demographic groups, including both genders [36–41]. Given the importance of these factors, the measurement and assessment of social support are critical for understanding and promoting PA participation across various cultures and populations. However, Chinese versions of measurement tools related to social support for exercise are limited and confounding.

Some Hong Kong scholars developed a set of questionnaires for children that measure environmental and psychosocial-related factors that influence PA participation [42], but this self-developed scale focused more on the environmental factors. Another study conducted by Liang et al. [43], translated a set of scales used to measure PA participation correlates, which included a different version of a 10-item social support scale, received good psychometric support among Hong Kong Chinese children. As for mainland China, only one study has translated a simplified version of the scale into Mandarin in mainland China [18]. This study simplified the 20 items in the original scale to six items and tested good validity in adults. It is worth noting that the simplified version of the 6-item scale may not provide comprehensive perspectives for relevant research. These existing scales exhibit variations in both content and the validated populations, and a lack of adequate supporting research. Conversely, the validated version of the social support scale for exercise in this paper, which was updated by Yang et al. (2005) [25], has received several supporting studies in different countries [3, 44]. Therefore, our study is warranted.

In the present study, researchers proposed a standardized measure consistent with prior research that allows us to understand better the social factors that may influence and enhance PA behaviors among Chinese children and adolescents. This study reveals that the Chinese version of the social support scale for exercise is adequate and compatible with prior research when evaluating PA participation behaviors among Chinese adolescents. Since all items were retained, the scale was determined to have acceptable validity and reliability, and its implementation will provide researchers, educators, and health practitioners with a reliable tool to assess and understand the dynamics of social support in the context of PA among Chinese adolescents.

### Strength and limitations

In our study, we employed a substantial sample size to examine the psychometrics of the Chinese version of the social support scale for exercise, along with a smaller sample to assess the test-retest reliability of the scale. Nonetheless, certain limitations should be acknowledged that may have influenced the findings. Firstly, it’s worth noting that our survey encompassed participants from a single city, which could temper the extent to which we can apply these findings more broadly. Nevertheless, the sizable nature of our study’s participant pool serves to bolster the robustness and implications of our conclusions. Secondly, our study leaned on a self-reported questionnaire, a method that comes with inherent limitations. The accuracy of data might be influenced by response bias. To mitigate this concern, all participants were assured of the confidentiality of their responses. We urged them to provide sincere answers and not to discuss their responses with peers. Moreover, to tackle this issue more effectively, future investigations might consider incorporating probability sampling techniques to enhance the overall applicability of the results. Furthermore, we did not assess criterion-related validity and convergent validity by comparing the SE-C scale with other related measures. We recommend that researchers explore these aspects in future studies to further validate the scale and broaden its utility.

## Conclusion

This study provided psychometric support for using the Chinese version of the social support for exercise scale among Chinese primary and middle school students. The Chinese version of the social support for exercise scale was valid and reliable for Chinese children and adolescents. All items were retained and validated as appropriate for the sample data. Our findings may be helpful to policymakers, professionals, and exercise educators in assessing and implementing necessary programs to endorse and raise a better understanding of the value of regular physical activities.

### Implications for future studies

The extent to which these scales correlate with PA in a more representative sample of Chinese people of different ages, educational levels, occupations, and health statuses are needed. Tests of the sensitivity of these scales to change over time through longitudinal studies are also important.
